# Shigellosis Presenting as Meningism

**DOI:** 10.7759/cureus.14707

**Published:** 2021-04-27

**Authors:** Monarch V Shah, Oluwatofunmi Olowoyo, Sanya Chandna, Ira Gurland

**Affiliations:** 1 Internal Medicine, Saint Peter’s University Hospital, New Brunswick, USA; 2 Internal Medicine, St George's University, New Brunswick, USA; 3 Infectious Diseases, Saint Peter’s University Hospital, New Brunswick, USA

**Keywords:** shigellosis, meningitis pain, strongyloides hyperinfection syndrome

## Abstract

*Shigella* is a common cause of gastroenteritis and can also lead to serious complications such as dehydration, seizures, hemolytic uremic syndrome, and neurological complications. In this paper, we describe a case of a 39-year-old man who was admitted septic, with altered mental status and gastrointestinal symptoms. During the evaluation, he was found to have positive meningeal signs, non-anion gap metabolic acidosis, enteric panel positive for *Shigella flexneri*, positive IgG for *Strongyloides*, and pancolitis on computed tomography (CT) of the abdomen and pelvis. He was treated for infectious colitis and initially treated empirically for meningitis, but antibiotics were later discontinued due to rapid improvement of meningeal signs. To reduce the risk of disseminated infection, the patient was also treated for *Strongyloides*.

## Introduction

*Shigella* is a common cause of bacterial diarrhea transmitted by direct person-to-person spread, contaminated food, and water. Seizures are the most common neurologic complication associated with *Shigella* infection and they occur almost exclusively in children younger than 15 years [1,2]. Other neurologic findings such as encephalopathy with lethargy, confusion, and headache have been described in children but rarely in adults [3]. Herein, we describe a case of shigellosis presenting with meningism and an incidental lesion of neurocysticercosis.

## Case presentation

**Figure 1:**
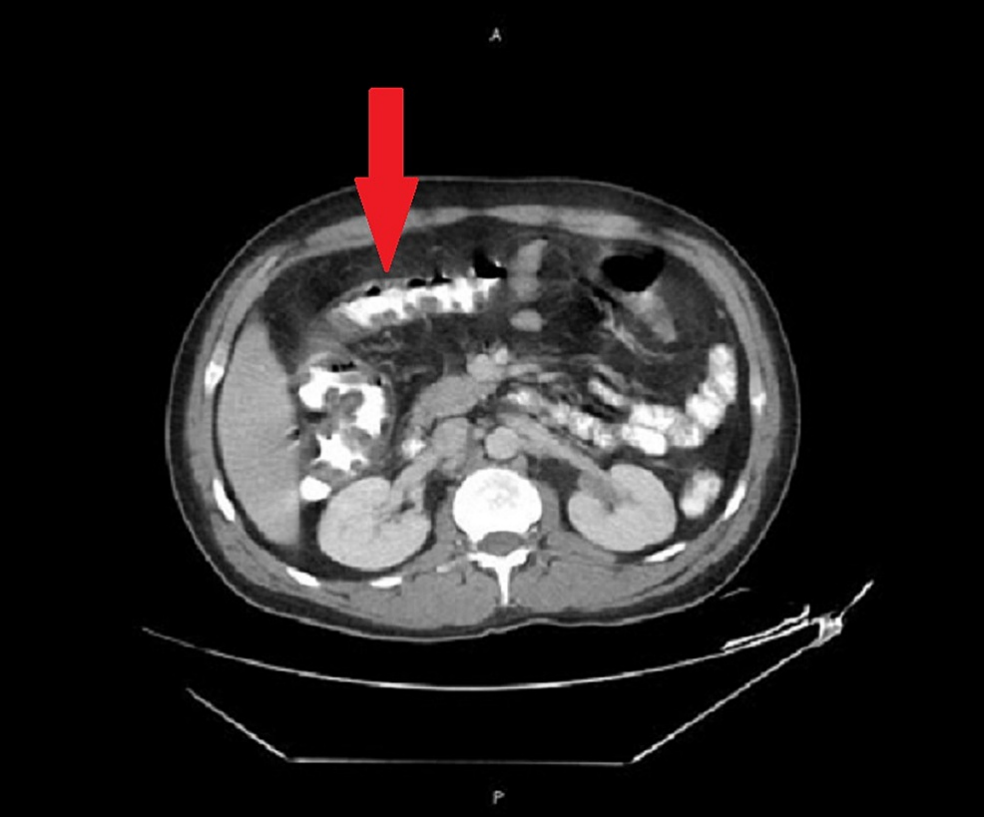
CT of the abdomen and pelvis with contrast showing diffuse colonic wall thickening from the rectosigmoid to the proximal ascending colon consistent with pancolitis.

A 39-year-old Mexican man with a history of a seizure 14 years ago was brought to the emergency department (ED) with a complaint of one day of confusion. Two days before admission, he had approximately 10 bouts of watery, non-bloody stools. Additionally, he had four episodes of non-bloody, non-bilious vomiting. There was associated abdominal pain, dizziness, and lightheadedness. On the day before admission, he developed fever, chills, myalgias, and arthralgias followed by a 10/10 throbbing headache with neck stiffness. His wife noticed he was confused, and thus she brought him to the ED for evaluation. His last meal two days ago was tamales with pork. No other family members consumed that food. There were no sick contacts at home or work. He reported no tick bites. His past medical history was significant for one episode of seizure 14 years ago, the etiology of which was unknown, and he was not treated with anti-epileptic medications. He was born and raised in Mexico. He moved to the USA at the age of 15 years. He had not traveled recently. He is married and has no promiscuous sexual activity. He works in construction and denies smoking, consuming alcohol, or recreational drug use. On examination, his temperature was 103.1°F, pulse was 132 beats per minute, blood pressure was 106/71 mm Hg, respiratory rate 18 breaths per minute, and 98% oxygen saturation on room air. Overall, the patient was in significant distress. He was oriented to time place and person, had a dry oral mucosa, and had a positive jolt test. On abdominal examination, there was diffuse generalized abdominal tenderness and hyperactive bowel sounds, with no focal neurological deficits. White cell count was 13.1 x 10^3^ mm^3^ (normal range: 4.0-11.0 x 10^3^ mm^3^), and the basic metabolic panel was significant for non-anion gap metabolic acidosis. *Clostridium difficile* toxin by polymerase chain reaction (PCR), and *Cryptosporidium* and *Giardia* antigens were negative. Influenza A and B and respiratory viral panel by PCR were negative. Rapid HIV1 and HIV2 screens were non-reactive. CT of the abdomen and pelvis (Figure 1) showed diffuse colonic wall thickening from the rectosigmoid to the proximal ascending colon consistent with pancolitis.

**Figure 2:**
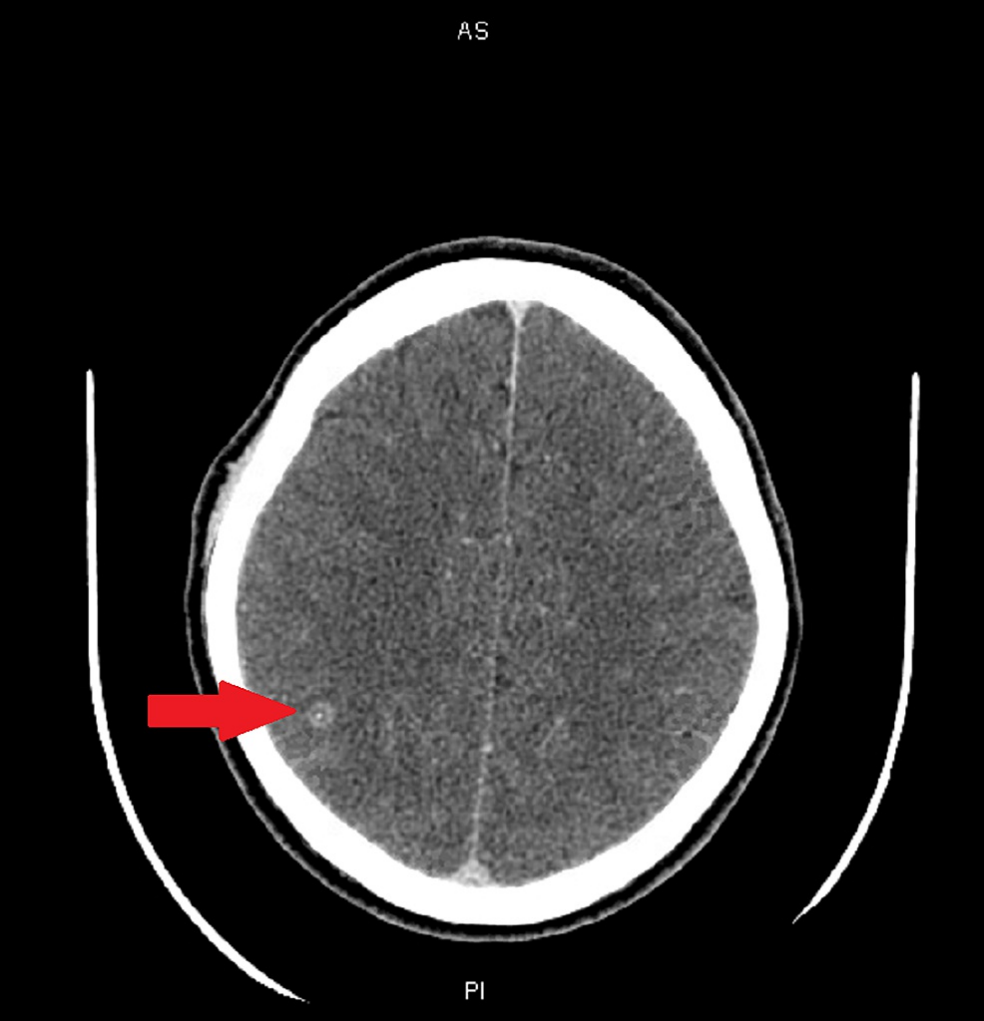
CT of the head without contrast showing a 7-mm ring-enhancing lesion with a dot-like intrinsic calcification along the right parietal lobe.

CT of the head (Figure 2) showed a single 7-mm ring-enhancing lesion with a dot-like intrinsic calcification along the right parietal lobe.

**Figure 3:**
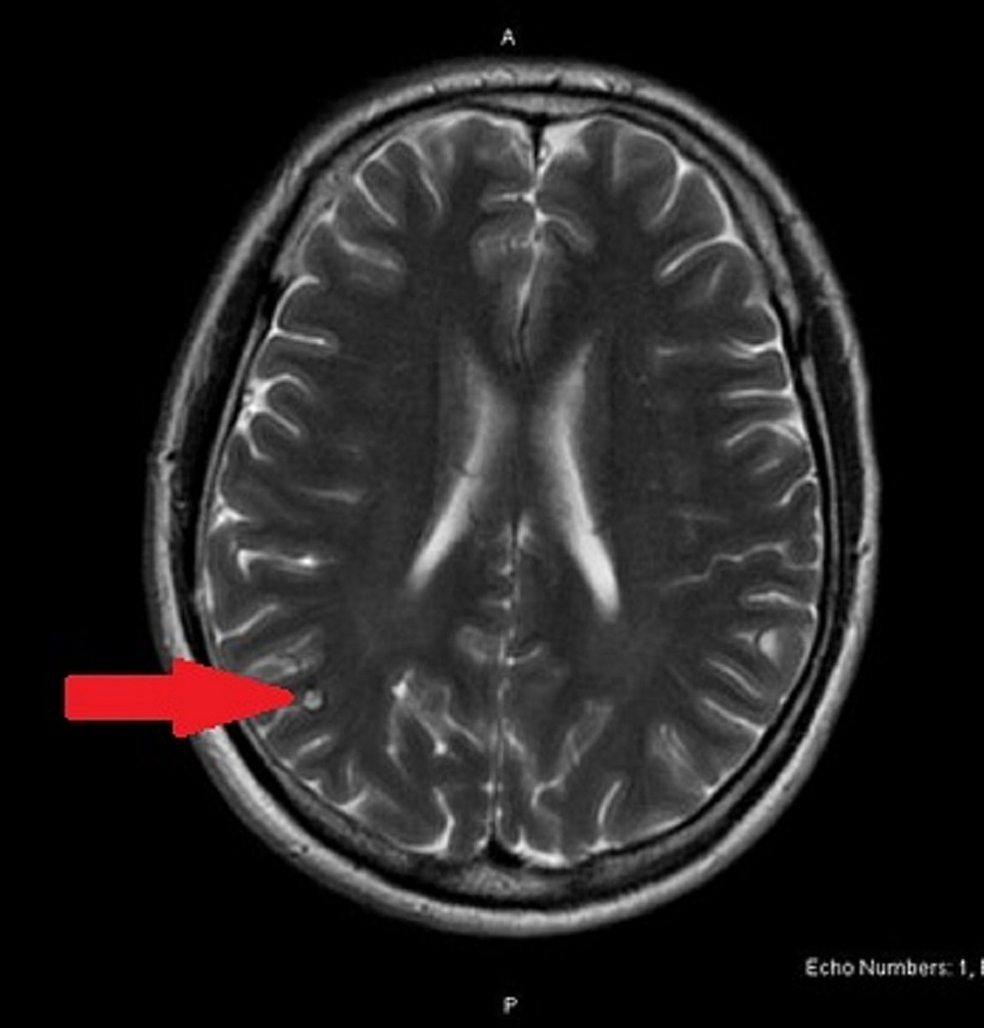
MRI of the brain with contrast showing a 5-mm T2 hyperintense, homogeneously enhancing lesion in the right parietal lobe with associated calcification.

His physical findings were suggestive of meningitis, and therefore he was treated empirically with vancomycin and ceftriaxone. He received 30 mL/kg bolus of fluid resuscitation. Our differential diagnosis at that point was sepsis from meningitis versus bacterial gastroenteritis. The enteric panel was PCR-positive for *Shigella*. Stool culture was positive for *Shigella flexneri*. Magnetic resonance imaging (MRI) of the brain (Figure 3) showed a small T2 hyperintense, homogeneously enhancing lesion in the right parietal lobe with associated calcification.

After empiric treatment for bacterial meningitis with IV vancomycin and ceftriaxone, neck stiffness resolved within 8-10 hours of admission, and meningitis was thought to be less likely. A lumbar puncture was deferred and vancomycin was discontinued. The abdominal pain was attributed to *Shigella flexneri* infectious colitis and was treated with IV ceftriaxone and azithromycin. The patient's MRI of the brain demonstrated a small lesion with some calcification. This was considered to be an incidental finding. The decision at that time was to observe and not treat. An ophthalmology evaluation demonstrated no retinal lesions. Before considering treatment, a QuantiFERON assay and *Strongyloides* immunoglobulin G (IgG) were sent. The QuantiFERON assay was negative. The *Strongyloides* IgG was positive and the patient was treated with two doses of ivermectin (0.200 mg/kg daily for two days). However, given that the patient was asymptomatic and the lesion was partially calcified, we decided to hold treatment for neurocysticercosis.

## Discussion

*Shigella* is a rare cause of invasive infections such as meningitis or encephalopathy in adults. Although fairly common in infants, children, or immunocompromised patients; clinical presentation with severe neurologic symptoms is rarely seen in adults. One article from Spain reported on a similar case with *Shigella flexneri* enteritis [4]. The second case of shigellosis-associated encephalopathy is described in a patient with *Shigella sonnei* gastrointestinal infection [5]. In adults, most reported cases of meningitis secondary to shigellosis have been seen in patients with *Shigella flexneri*, *Shigella sonnei*, and *Shiga* toxin-producing *Escherichia coli* [6]. These cases present similarly with fever, altered mental status, and bloody diarrhea, nausea, and vomiting. Seizures are also a presenting feature of this disease. Although previous studies have postulated a connection between *Shiga* toxin and encephalopathy, there is no clear evidence to support this. As a result, there is a need for further investigation into the pathogenicity behind *Shigella* meningitis.

Hyperinfection syndrome from strongyloidiasis occurs mainly in immunocompromised patients and those on steroids or immunosuppressive drugs and need prompt treatment to prevent disseminated infection [7]. Intraparenchymal cysts in neurocysticercosis may present with seizures and symptoms of raised intracranial pressure [8]. However, most patients are usually asymptomatic for many years and may eventually develop calcified lesions [9].

In our case, the patient had a history of seizures 14 years ago and an incidental MRI finding of neurocysticercosis. Given the patient's history and presentation, the calcified cysts were most likely a result of an old infection.

## Conclusions

Shigellosis is a common disease worldwide. It causes bloody stool with mucus, together with fever and abdominal cramps, but it can also present with meningitis. Antimicrobial therapy can lower the overall mortality rate and shortens the duration of gastrointestinal complaints. Just like the patient in our report, initially patients might be suspected of having bacterial meningitis or viral encephalitis and get treated accordingly. Calcified lesions of neurocysticercosis do not warrant treatment, and screening for *Strongyloides* is done of at-risk patients to avoid hyperinfection syndrome in patients treated with corticosteroids. Also, in our case, there was some initial controversy about whether to treat neurocysticercosis. Once it became clear that this was an incidental finding, treatment was deferred.
